# A light and sound show of cancer therapy

**DOI:** 10.1038/s42003-024-05900-8

**Published:** 2024-02-20

**Authors:** Sharmistha Chatterjee

**Affiliations:** Division of Molecular Medicine, Bose Institute, Kolkata, India

## Abstract

Nanoparticle therapy continues to be an attractive avenue of targeted and personalised therapies. A molecular nano-conjugate developed by Zeng et al. effectively targets cancer cells and aids in their diagnosis, therapy, and also optimises innate immune responses.

Cancer cells thrive in an environment that is optimal for their growth. It is called the tumor microenvironment and is characterised by many specific biochemical and biophysical parameters^1,2^, including the presence of a specific type of immune cells that maintain a local immune homeostasis. These regulatory T-cells (or T-reg cells) aid in cancer progression, resulting in poor prognosis of the cancerous state by any immunotherapeutic modalities^3^. They prevent immune responses by other cells like effector and cytotoxic T-cells, B cells, natural killer cells, macrophages, etc., towards cancer cells. Glioblastoma multiforme, an intracranial tumor type, clearly shows this immune-inhibitory effect of T-reg cells. Interestingly, IL-2R and CD25 proteins are always expressed on T-reg cells, which make them effective targets for antibody-conjugated targeted therapies^4,5^. Despite this, specific elimination of the immunosuppressive T-reg cells lodged at the cancer site while saving the body’s normal immune homeostasis has been an ongoing challenge to achieve effective cancer therapy.

Recently, Zeng et al. devised a nanoparticle (PEG/αCD25-Cy7/TMZ) that responds to ultrasound-assisted laser irradiation, commonly called photoacoustic imaging, to diagnose and treat glioblastoma more effectively^6^. It contains a specific molecule (Cy7) in its preparation that, under photoacoustic imaging, sends out very specific signals pertaining to its immediate space, that can be then imaged to provide a clear view of the space around the location of the particle, ranging from less than 1 mm to more than 10 cm area within the tissue. This method does not have as strong penetrative properties as MRI or CT, but can still produce an image to a depth up to 45 microns. To the nanoparticle, the authors also attached antibodies targeted towards those T-reg cells that had already lodged themselves in the tumor microenvironment. Thus, the authors found a way to effectively diagnose the cancer at minute resolutions in the brain tissue as well as specifically eliminate the T-reg cells present at the cancer site. The nanoparticles are responsive to GSH, a cellular antioxidant found in very high levels inside the cancer cells as well as the tumor microenvironment, which makes the release of the Cy7 and the loaded anticancer drug temozolomide or TMZ, very much specific in its area of action. Elimination of the T-reg cells in the tumor microenvironment then aids in the increased activity of the immunotherapy drug IDO towards the cancer, and sensitizes the tumor site to other immune cell actions. The targeted drug-loaded nanoparticles increased cellular death while photoacoustics showed the exact location of the cancer cells within the brain tissue, and how it changed over time, with increased immune responses from the body.

This study comes in the line of a long-standing struggle against cancer, and specifically glioblastoma, to devise effective therapeutic strategies. Photo-acoustic imaging is non-invasive and less severe than the currently accepted diagnostic protocols. Additionally, the nanoparticle also kills the cancer cells in which it is taken up, and makes way for the normal cellular and humoral immunity of the body to act without hindrance. It paves the way for devising better strategies for immunotherapy in cancer, and its diagnostics. As a cancer theranostic, this nanoparticle system has great potential for future clinical trials.
